# Effects of nitrogen additions on mesophyll and stomatal conductance in Manchurian ash and Mongolian oak

**DOI:** 10.1038/s41598-020-66886-x

**Published:** 2020-06-22

**Authors:** Kai Zhu, Anzhi Wang, Jiabing Wu, Fenghui Yuan, Dexin Guan, Changjie Jin, Yushu Zhang, Chunjuan Gong

**Affiliations:** 1 Key Laboratory of Forest Ecology and Management, Institute of Applied Ecology, Chinese Academy of Sciences, Shenyang, 110016 China; 2 University of Chinese Academy of Sciences, Beijing, 100049 China; 3 The Institute of Atmospheric Environment, China Meteorological Administration, Shenyang, 110166 China

**Keywords:** Ecology, Forest ecology, Plant ecology, Plant physiology

## Abstract

The response of plant CO_2_ diffusion conductances (mesophyll and stomatal conductances, *g*_m_ and *g*_sc_) to soil drought has been widely studied, but few studies have investigated the effects of soil nitrogen addition levels on *g*_m_ and *g*_sc_. In this study, we investigated the responses of *g*_m_ and *g*_sc_ of Manchurian ash and Mongolian oak to four soil nitrogen addition levels (control, low nitrogen, medium nitrogen and high nitrogen) and the changes in leaf anatomy and associated enzyme activities (aquaporin (AQP) and carbonic anhydrase (CA)). Both *g*_m_ and *g*_sc_ increased with the soil nitrogen addition levels for both species, but then decreased under the high nitrogen addition level, which primarily resulted from the enlargements in leaf and mesophyll cell thicknesses, mesophyll surface area exposed to intercellular space per unit leaf area and stomatal opening status with soil nitrogen addition. Additionally, the improvements in leaf N content and AQP and CA activities also significantly promoted *g*_m_ and *g*_sc_ increases. The addition of moderate levels of soil nitrogen had notably positive effects on CO_2_ diffusion conductance in leaf anatomy and physiology in Manchurian ash and Mongolian oak, but these positive effects were weakened with the addition of high levels of soil nitrogen.

## Introduction

Nitrogen (N) is an important nutrient for plant photosynthesis because it alters N allocation between photosynthetic components^1^, but excessive nitrogen depositions break the soil nitrogen balance and have a strongly negative effect on photosynthesis^2,3^. With global climate change, nitrogen deposition has increased dramatically worldwide^4,5^, and its effects on CO_2_ diffusion conductance (both mesophyll and stomatal conductances, *g*_m_ and *g*_sc_) in photosynthesis have attracted considerable attention in global change, physiological ecology, plant physiology and other fields^6–8^. In contrast to *g*_sc_, *g*_m_ has often been neglected in previous studies, with its supply being assumed to be unlimited^9,10^; the importance of *g*_m_ is only being highlighted in recent decades with the advent of advanced instruments and measuring technologies, and studies of *g*_m_ have also increased correspondingly^1,11–14^.

The relationship of *g*_sc_ with soil nitrogen has been widely explored; *g*_sc_ increased with soil nitrogen additions overall^15–17^, and *g*_m_ also showed a positive correlation with moderate soil nitrogen supplementation in general^1,6,7^. However, excessive nitrogen application resulted in a decrease in the ability to scavenge reactive oxygen species (ROS) in wheat^18^. Many changes under excessive nitrogen application may also occur in photosynthesis electron transport rate (*J*_f_) or actual photochemical efficiency of photosystem II (*Φ*_PS_ II). In addition, several recent papers also reported that high N conditions reduced evapotranspiration, which resulted in constraining N uptake in almond trees^18–20^, and this seemed related to the slight decrease in *g*_sc_ and leaf N content under high nitrogen condition. Hence, it has not been determined how *g*_m_ and *g*_sc_ responded to excessive nitrogen additions (≥69 kg N ha^−1^ a^−1^). Besides, the changes in leaf anatomy and associated physiological traits were both considered to be important mechanisms in determining *g*_m_ and *g*_sc_^14,21–29^.

Xiong *et al*.^14^ revealed that leaves with larger *g*_m_ in high nitrogen supplement had a larger leaf thickness (*T*_leaf_) and mesophyll surface area exposed to intercellular space per unit leaf area (*S*_mes_) than those in low nitrogen supplementation, and Zhu *et al*.^30^ also showed that *T*_leaf_ and mesophyll cell thickness (*T*_mes_) both imposed a positive effect on *g*_m_ recovery in Manchurian ash and Mongolian oak, while *S*_mes_ had a negative effect on this parameter. In addition, Xiong *et al*.^29^ and Zhu *et al*.^30^ both revealed the determination of stomatal features (size and density) and opening status (SS) to *g*_sc_, and Zhu *et al*.^30^ suggested a positive correlation between SS and *g*_sc_ in Manchurian ash and Mongolian oak, which was also supported by the study of Xu and Zhou (2008)^31^. Besides, the changes of *g*_m_ and *g*_sc_ would also be significantly affected by aquaporin (AQP) and carbonic anhydrase (CA) activities^14,21^. AQP mediates the genes in the plasma membrane intrinsic protein (PIP) aquaporin family, and its increase in activity strongly promotes the expression of PIPs and improves CO_2_ membrane permeability^32–34^. CA regulates *g*_m_ mainly by changing the dynamics of CO_2_ to ^−^HCO_3_^28^. However, it has not been determined how leaf anatomies and AQP and CA activities would change in the Changbai Mountains with the addition of various levels of soil nitrogen.

Manchurian ash (*Fraxinus mandshurica* Rupr.) and Mongolian oak (*Quercus mongolica* Fish. ex Ledeb), two species in Oleaceae and Fagaceae, respectively, are widely distributed in the Changbai Mountains, China. Their responses of CO_2_ diffusion conductance to soil nitrogen additions have rarely been reported; and the mechanisms governing *g*_m_ and *g*_sc_ in leaf anatomy and physiology have not been determined. Hence, we measured both *g*_m_ and *g*_sc_ and their related anatomical and physiological traits, including leaf N content and AQP and CA activities, to explore the responses of CO_2_ diffusion conductance to the addition of various levels of soil nitrogen. The present study will advance our mechanistic understanding of global nitrogen deposition impacts on carbon cycling in tree species.

## Results

### Effects of soil nitrogen additions on *g*_m_

**Figure 1:**
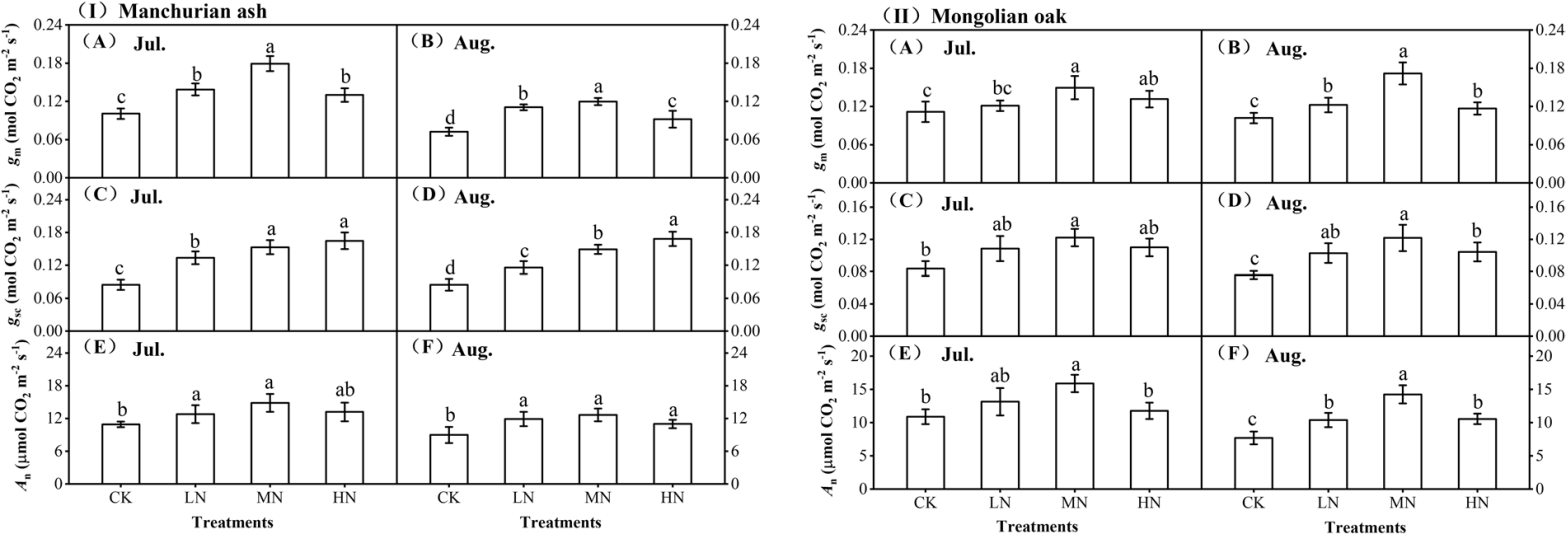
Changes of plant *g*_m_, *g*_sc_ and *A*_n_ with soil N additions in Manchurian ash (I) and Mongolian oak (II) in July and August. Values were means ± SE (n = 5), and different lowercase letters (**a**–**c**) indicated significant difference at *P* < 0.05. CK, the control; LN, low nitrogen addition; MN, medium nitrogen addition; HN, high nitrogen addition.

Considerable changes in *g*_m_ were observed in both species after soil nitrogen addition compared with the control values (CK) (Fig. 1a,b). The *g*_m_ gradually increased with the addition of various levels of soil nitrogen, reaching the maximum with the medium level of 46 kg N ha^−1^ a^−1^ (MN) and then decreasing with the addition of 69 kg N ha^−1^ a^−1^ (HN) while remaining greater than the *g*_m_ observed for the CK group. Overall, *g*_m_ showed significant differences among the four nitrogen-addition treatments in both species, in which it was significantly larger in the MN treatment than in the other treatments (*P* < 0.05). In addition, the *g*_m_ in August was slightly lower than that in July in both species overall.

### Effects of soil nitrogen additions on *g*_sc_

Concurrently, soil nitrogen addition also resulted in a significant increase in *g*_sc_, with a different trend being observed between Manchurian ash and Mongolian oak (Fig. 1c,d). In Manchurian ash, *g*_sc_ continued to increase with nitrogen addition in July and August, but it increased from low nitrogen (LN, 23 kg N ha^−1^ a^−1^) to MN and then decreased with the addition of HN in Mongolian oak. The *g*_sc_ with the nitrogen addition treatments was significantly larger than that of the control values in July and August in both species (*P* < 0.05). Overall, the *g*_sc_ in Manchurian ash was considerably larger than that in Mongolian oak. In addition, influenced by the changes in *g*_m_ and *g*_sc_, leaf *A*_n_ also increased first and then decreased with the addition of progressively higher levels of soil nitrogen (Fig. 1e,f), indicating that plant photosynthetic capacities could also be considerably strengthened by soil nitrogen additions.

### Effects of soil nitrogen addition on leaf anatomical characteristics

#### Effects on mesophyll anatomical traits

The addition of soil nitrogen caused some changes in mesophyll anatomical traits (Table 1), including leaf (*T*_leaf_) and mesophyll cells (*T*_mes_) thicknesses, the surface area of mesophyll cells exposed to intercellular space (*S*_mes_) and the ratio of mesophyll surface (*A*_mes_) to total leaf surface area (*A*_T_) corrects for the actual area available for CO_2_ diffusion (*A*_T_/*A*_mes_). *T*_leaf_, *T*_mes_, *S*_mes_ and *A*_T_/*A*_mes_ all increased after soil nitrogen addition in overall without *T*_leaf_ in HN and *A*_T_/*A*_mes_ in LN treatment and were lower than the CK values. When nitrogen supplementation was no more than 69 kg N ha^−1^ a^−1^, *T*_leaf_ and *S*_mes_ increased with nitrogen addition in both species, and *T*_mes_ also showed increases with the addition of soil nitrogen in Manchurian ash and reached a maximum in the MN treatment, while it reached a maximum in the LN treatment in Mongolian oak. Significant differences between nitrogen addition treatments were observed in *T*_leaf_ and *T*_mes_, while no significant differences were shown for *S*_mes_ and *A*_T_/*A*_mes_ overall in both species (*P* < 0.05).

**Table 1:** Values of leaf anatomical characteristics for different nitrogen addition treatments in Manchurian ash and Mongolian oak.

	Manchurian ash						Mongolian oak					
	*T*_leaf_ (μm)	*T*_mes_ (μm)	*S*_mes_ (μm^2^ μm^−2^)	*A*_T_/*A*_mes_	*g*_ias_ (10^−2^ m s^−1^)	*g*_liq_ (10^−4^ m s^−1^)	*T*_leaf_ (μm)	*T*_mes_ (μm)	*S*_mes_ (μm^2^ μm^−2^)	*A*_T_/*A*_mes_	*g*_ias_ (10^−2^ m s^−1^)	*g*_liq_ (10^−4^ m s^−1^)
CK	130.1 ± 11.0^bc^	116.3 ± 5.1^bc^	12.1 ± 2.0^a^	1.91 ± 0.08^ab^	4.47 ± 0.37^d^	1.08 ± 0.06^b^	206.8 ± 3.2^bc^	158.1 ± 6.7^c^	12.9 ± 1.3^b^	1.81 ± 0.11^a^	5.41 ± 0.33^c^	1.14 ± 0.06^b^
LN	135.9 ± 5.1^b^	122.9 ± 4.2^ab^	14.2 ± 3.8^a^	1.74 ± 0.11^b^	11.93 ± 0.79^a^	1.19 ± 0.14^ab^	213.7 ± 4.7^b^	185.9 ± 3.5^a^	13.0 ± 1.5^ab^	1.67 ± 0.40^a^	6.21 ± 0.42^b^	1.24 ± 0.12^ab^
MN	148.2 ± 7.3^a^	130.2 ± 2.5^a^	15.2 ± 2.6^a^	2.35 ± 0.53^a^	10.13 ± 0.74^b^	1.28 ± 0.05^a^	227.4 ± 6.0^a^	171.2 ± 8.0^b^	16.0 ± 2.0^a^	1.85 ± 0.34^a^	7.22 ± 0.22^a^	1.32 ± 0.07^a^
HN	122.9 ± 3.6^c^	112.9 ± 3.3^c^	18.0 ± 5.0^a^	2.43 ± 0.16^a^	8.11 ± 1.03^c^	1.06 ± 0.07^b^	202.9 ± 2.4^c^	167.1 ± 4.1^bc^	14.4 ± 1.7^ab^	1.78 ± 0.22^a^	5.76 ± 0.59^bc^	1.17 ± 0.04^b^

All data were means ± SE (n = 5). Different lowercase letters (a, b, c) indicated significant differences between nitrogen addition treatments at *P* < 0.05. CK, the control; LN, low nitrogen addition; MN, medium nitrogen addition; HN, high nitrogen addition.

#### Changes of leaf g_ias_ and g_liq_

Changes of *T*_mes_ and *A*_T_/*A*_mes_ to soil nitrogen additions also directly caused changes in leaf gas-phase (*g*_ias_) and liquid-phase CO_2_ diffusion conductance (*g*_liq_) inside mesophyll cells (Table 1). Both *g*_ias_ and *g*_liq_ gradually increased with MN and then decreased under HN treatment. A significant difference between nitrogen addition treatments was observed in *g*_ias_, while *g*_liq_ did not change significantly overall (*P* < 0.05).

#### Effects on stomatal parameters

Correspondingly, soil nitrogen addition also caused considerable changes in stomatal parameters, mainly including stomatal pore length and width at the centre of the stoma (PL and PW), stomatal opening status (SS) and density (*D*_s_) (Table 2). As two important components to SS, PL and PW both increased with the MN treatment and were similar in both species, but in the HN treatment, they both fell below the CK values. Consequently, leaf SS gradually increased with soil nitrogen addition and then significantly decreased to a minimum under HN treatment. Besides, significant increases also happened to leaf *D*_s_ after soil nitrogen additions (*P* < 0.05) (Table 2), in which Manchurian ash showed a gradually increased *D*_s_ with soil nitrogen addition, reaching the maximum under HN treatment, while *D*_s_ increased first then decreased in Mongolian oak, reaching its maximum under LN treatment.

**Table 2:** Values of stomatal parameters for different nitrogen addition treatments in Manchurian ash and Mongolian oak. All data were means ± SE (n = 64).

	Manchurian ash				Mongolian oak			
	PL (μm)	PW (μm)	SS (μm^2^)	*D*_s_ (10^−2^ N cm^−2^)	PL (μm)	PW (μm)	SS (μm^2^)	*D*_s_ (10^−2^ N cm^−2^)
CK	7.01 ± 0.70^b^	3.04 ± 0.97^ab^	17.57 ± 1.72^b^	6.32 ± 0.45^b^	6.36 ± 0.52^a^	2.47 ± 0.58^b^	12.78 ± 1.15^b^	9.48 ± 0.45^b^
LN	7.26 ± 0.14^ab^	3.18 ± 0.27^a^	18.10 ± 1.43^ab^	8.28 ± 0.94^a^	6.53 ± 0.19^a^	2.54 ± 0.35^b^	13.06 ± 2.20^b^	11.89 ± 0.69^a^
MN	7.66 ± 0.19^a^	3.28 ± 0.27^a^	19.74 ± 2.11^a^	8.43 ± 1.14^a^	6.71 ± 0.30^a^	3.13 ± 0.12^a^	16.52 ± 1.42^a^	11.14 ± 0.26^a^
HN	7.35 ± 0.05^ab^	2.73 ± 0.13^b^	15.74 ± 0.68^b^	9.93 ± 0.90^a^	6.25 ± 0.29^a^	2.40 ± 0.19^b^	11.81 ± 1.51^b^	9.63 ± 0.69^b^

Different lowercase letters (a, b, c) indicated significant differences between nitrogen addition treatments at *P* < 0.05. SS, stomatal opening status; PL and PW, stomatal pore length and width at the centre of the stoma; *D*_s_, stomatal density. CK, the control; LN, low nitrogen addition; MN, medium nitrogen addition; HN, high nitrogen addition.

### Effects of soil nitrogen additions on leaf N content

Changes in leaf N content in both species during soil nitrogen additions are shown in Table 3. The leaf N content was enlarged by soil nitrogen addition and increased with medium nitrogen addition to MN and then decreased under HN treatment. Overall, no significant differences in leaf N content between soil nitrogen addition treatments were observed in both species (*P* < 0.05), and Mongolian oak saplings maintained a higher leaf N content in nitrogen addition treatments than did Manchurian ash saplings.

**Table 3:** Values of leaf N content (g kg^−1^) during soil nitrogen additions in July and August in both species.

	Manchurian ash		Mongolian oak	
	July	August	July	August
CK	13.1 ± 0.88^ab^	10.4 ± 0.31^b^	13.0 ± 1.22^b^	10.2 ± 0.93^b^
LN	14.4 ± 0.83^b^	11.8 ± 0.55^a^	16.4 ± 3.05^ab^	15.7 ± 2.73^a^
MN	15.2 ± 0.46^a^	11.9 ± 0.32^a^	19.1 ± 1.12^a^	15.5 ± 0.47^a^
HN	15.0 ± 1.85^ab^	11.1 ± 1.10^ab^	16.0 ± 1.48^ab^	12.8 ± 0.93^ab^

All values were means ± SE (n = 3). Different lowercase letters (a, b, c) indicated significant differences between nitrogen addition treatments at *P* < 0.05. CK, the control; LN, low nitrogen addition; MN, medium nitrogen addition; HN, high nitrogen addition.

### Effects of soil nitrogen addition on AQP and CA activities

Soil nitrogen addition also had great effects on leaf AQP and CA activities in both species (Table 4). In this study, the activities of AQP and CA were both enlarged after soil nitrogen addition, and they also gradually strengthened with soil nitrogen added to MN and then weakened with the HN treatment. Similarly, the activities of these two enzymes also did not show significant differences between the four nitrogen addition treatments overall (*P* < 0.05).

**Table 4:** Values of leaf AQP and CA activities during soil nitrogen additions in both species.

	Manchurian ash		Mongolian oak	
	AQP (U g^−1^)	CA (U g^−1^)	AQP (U g^−1^)	CA (U g^−1^)
CK	5.57 ± 0.29^b^	1.54 ± 0.09^a^	5.57 ± 0.15^c^	1.42 ± 0.10^ab^
LN	5.78 ± 0.09^ab^	1.57 ± 0.05^a^	6.00 ± 0.11^b^	1.40 ± 0.03^ab^
MN	5.92 ± 0.04^a^	1.64 ± 0.08^a^	6.80 ± 0.06^a^	1.51 ± 0.02^a^
HN	5.91 ± 0.13^a^	1.61 ± 0.09^a^	6.17 ± 0.31^b^	1.38 ± 0.03^b^

All values were means ± SE (n = 3). Different lowercase letters (a, b, c) indicated significant differences between nitrogen addition treatments at *P* < 0.05. CK, the control; LN, low nitrogen addition; MN, medium nitrogen addition; HN, high nitrogen addition.

## Discussion

### Increases in *g*_m_ and *g*_sc_ mostly resulted from the improvement of leaf N content

**Figure 2:**
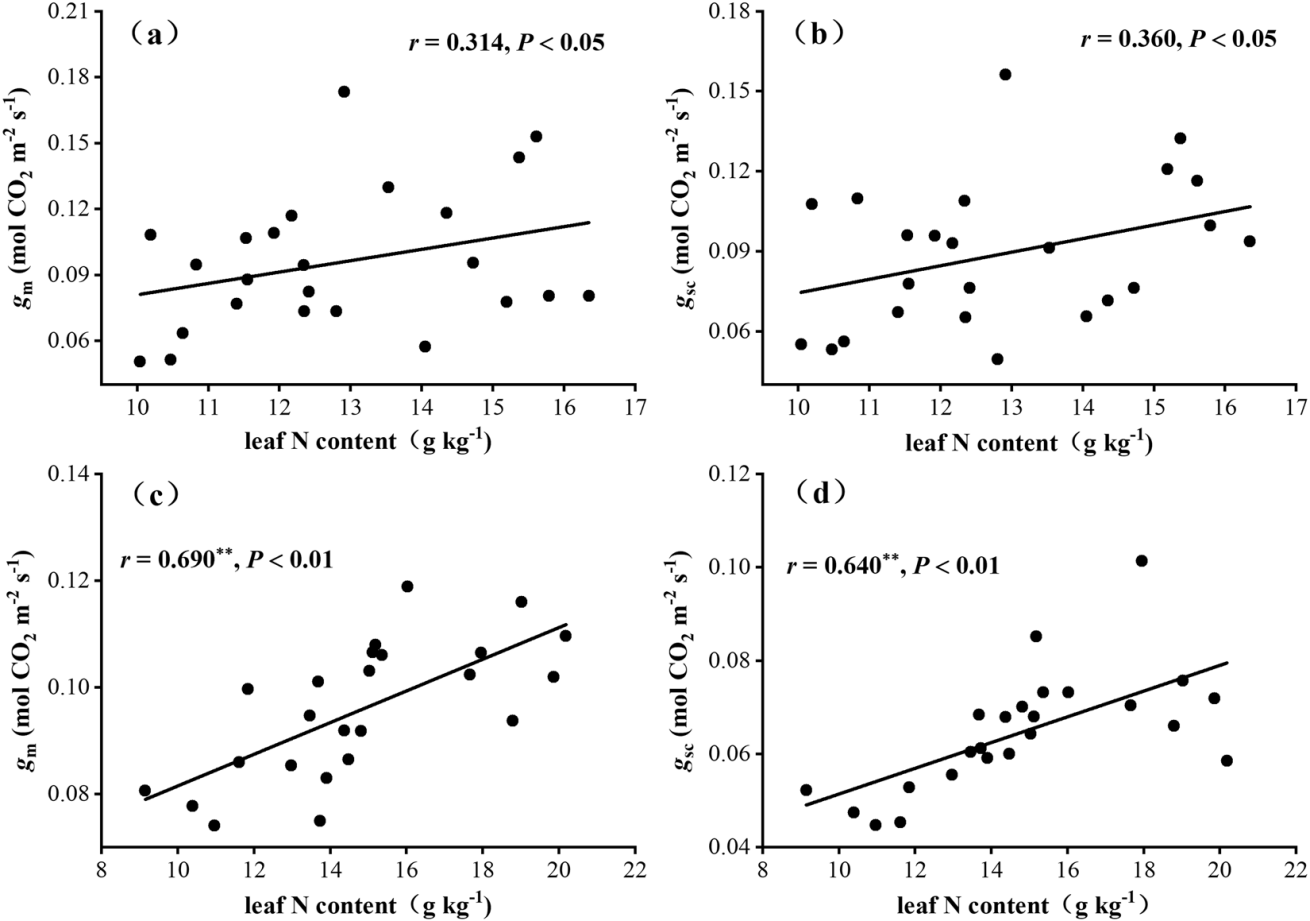
The correlations between *g*_m_, *g*_sc_ and leaf N content in Manchurian ash (**a**,**b**) and Mongolian oak (**c**,**d**). The coefficient of correlation (*r*) and significant correlation were at *P* < 0.05 and **P < 0.01.

Our results showed that both *g*_m_ and *g*_sc_ were enlarged by soil nitrogen addition; specifically, these values increased with nitrogen addition from LN to MN and then decreased with the addition of HN. Concurrently, leaf N content also presented a similar change with soil nitrogen addition (See Table 3). We believed that the increases of *g*_m_ and *g*_sc_ were largely related to the enlargement of leaf N content in this study because the relationships between leaf N content and *g*_m_ and *g*_sc_ strongly supported the promotion of leaf N content to *g*_m_ and *g*_sc_ increases, in which *g*_m_ and *g*_sc_ both showed a positive correlation with leaf N content, even though they were highly significant in Mongolian oak (*P* < 0.01) (Fig. 2). This finding was supported by the positive links between leaf N content and CO_2_ diffusion conductance (both *g*_m_ and *g*_sc_) in studies of Li *et al*.^6^ and Yamori *et al*.^1^.

The positive correlation between leaf N content and *g*_m_ found in the current study supported earlier observations that if leaves were treated individually, leaf N content explained 32% of measured variability in *A*_n_ but only 11% of that in *g*_m_, while variation in *g*_m_ explained 33% of variation in *A*_n_^47^. With three-quarters of leaf N associated with photosynthesis (mostly Rubisco and chlorophyll), the relationship between N and *g*_m_ might simply reflect the relationship between *A*_n_ and *g*_m_^48^. In this study, leaf N content increased under soil nitrogen addition conditions (Table 3), this made great improvements to leaf photosynthetic capacity and *g*_m_ (from CK to MN, see Fig. 1), but the excessive N supply (high N addition of 69 kg N ha^−1^ a^−1^, HN) would lower Rubisco activity and content, *g*_m_ and *C*_c_^49^.

On the other hand, the release of photorespiratory CO_2_ occurred in the bundle-sheath mitochondria might make an enriched CO_2_ partial pressure in the bundle-sheath chloroplast in C_3_ species and result in an increased amount of CO_2_ to be refixed by Rubisco^50–53^. The amount of (photo)respiratory CO_2_ that was refixed by Rubisco can be calculated from the diffusion resistances^54^. The *g*_m_, as an apparent conductance, could also be strongly affected by respiratory and photorespiratory CO_2_ diffusing towards the chloroplasts from the mitochondria^54^. In this study, both CO_2_ compensation point in the absence of respiration (Г^*^) and the mitochondrial respiration in the light (*R*_d_) differed among nitrogen addition levels in Manchurian ash and Mongolian oak (Table S2). This difference might enrich CO_2_ partial pressure in the bundle-sheath chloroplast in Manchurian ash and Mongolian oak, and increase the amount of CO_2_ refixed by Rubisco, finally making large influences on *g*_m_ responding to soil nitrogen additions. However, Г^*^ and *R*_d_ did not show significant declines under high nitrogen addition condition as other gas exchange parameters (Table S2). This might be due to the bias of the Laisk method in the estimation Γ*. The Laisk method was pointed out that what it estimated is the intercellular CO_2_ partial pressure (*C*_i_^*^), rather than the true CO_2_ photocompensation point^14,54^. In other words, the Г^*^ measured in the current study might be the *C*_i_^*^ actually, and this would lead to an enlarged CO_2_ photocompensation point under high nitrogen addition condition, but this only held under the assumption of combined resistance of chloroplast envelope and stroma (*r*_ch_) being negligible. If *r*_ch_ made up a significant portion of mesophyll resistance (*r*_m_), the *C*_i_^*^ was no longer necessarily smaller than the true Г^*^; it might be equal to Г^*^, or even greater^54^. Hence, the Г^*^ value estimated using the classical Laisk method and its response to soil N supplement might exist uncertainties under high or excessive nitrogen addition condition.

Since a positive relationship between leaf N content and CO_2_ assimilation was reported in numerous studies in which N increased Rubisco content and activity^1,6,55–59^, the positive effect of leaf N content on *g*_m_ and *g*_sc_ might also be achieved, as it improved the activities of related enzymes, such as AQP and CA^28,30,33^, which had been implicated in regulation of *g*_m_^48,60^. In this study, the activities of AQP and CA were strengthened by soil nitrogen addition (Table 4). This would largely promote the expression of genes greatly in the plasma membrane intrinsic protein (PIP) aquaporin family^34^. Furthermore, the diffusion of CO_2_ in mesophyll cells was not only a complexly physical process, but also a chemical reaction process^10^. The improvements of AQP and CA activities under nitrogen addition could stimulate the chemical reactions by improving the conversion of CO_2_ to ^−^HCO_3_ and the membrane permeabilities to CO_2_ at plasma membrane, cytosol and chloroplast stroma in mesophyll cells^28,32,61^. Additionally, nitrogen addition could also promote AQP gene expressions, like the PIP2 aquaporin gene family^62,63^, which benefited CO_2_ transmembrane transport and consequently increase *g*_m_^14^. Hence, the nitrogen addition in this study might result in an increased CO_2_ permeability in oocytes membranes expressing *Nt*AQP1^64^, eventually leading to the enhancements of *g*_liq_ and *g*_m_.

### Strong promotion of soil nitrogen addition on *g*_m_ and *g*_sc_, as explained in leaf anatomy

In this study, leaf anatomical structures were adjusted by soil nitrogen additions from the changes in anatomical characteristics (See Tables 1 and 2), which exhibited notably positive effects on *g*_m_ and *g*_sc_, as leaf anatomical structures were tightly correlated with *g*_m_ and *g*_sc_^14,26,56,61–63^. Furthermore, as two stages in CO_2_ diffusion, the gas- and liquid-phase diffusional processes were largely strengthened in nitrogen addition leaves, as suggested by the increases of *g*_ias_ and *g*_liq_ (See Table 1). However, *g*_ias_ and *g*_liq_ in the high nitrogen addition treatment were lower than those in the low and medium nitrogen addition treatments (LN and MN) in Manchurian ash and Mongolian oak, which was in keeping with the results obtained for rice in Li *et al*.^6^.

Both *T*_leaf_ and *T*_mes_ increased with soil nitrogen added to MN level and then decreased with the addition of HN (See Table 1); these treatments enlarged total leaf (*A*_T_) and mesophyll surface areas (*A*_mes_) for the actual areas available for CO_2_ diffusion (data not shown), widened CO_2_ flow pathways^44,56^, and correspondingly enlarged *g*_ias_. However, *g*_ias_ was often suggested to be the minor component to *g*_m_, while *g*_liq_ was the major one^38,42,64^. Hence, suggested by our previous conclusion that the recovery of *g*_m_ after soil rewatering was mainly resulted from the increase of *g*_liq_ in Zhu *et al*.^30^, we believed the important promotion of soil nitrogen addition on *g*_m_ increase in this study would also be reached from the enlargement in *g*_liq_. The *g*_liq_ is tightly related to *S*_mes_, cell wall thickness (*T*_cw_) and the chloroplast surface facing the intercellular space per unit leaf area (*S*_c_)^24,26,42,65^. The increase of *S*_mes_ in this study (See Table 1) enlarged the touching area between CO_2_ and mesophyll cells and then improved the efficiency of CO_2_ transmembrane diffusion. Although we did not measure *T*_cw_ and *S*_c_, the *S*_c_ should be enlarged, while *T*_cw_ decreased, by soil nitrogen addition in this study, as *S*_c_ had been revealed to increase with nitrogen supply in rice^14^, and *T*_cw_ was negative with both *S*_c_ and *g*_m_^66,67^. The changes in *S*_c_ and *T*_cw_ would also strongly promote increased *g*_m_. Finally, the increase of *A*_T_/*A*_mes_ caused by mesophyll anatomical changes directly resulted in the enlargement of *g*_liq_. In addition, leaf PL and PW were enlarged by soil nitrogen additions in both species (See Table 2), which resulted in the improvement of stomatal opening status (SS) and broadened CO_2_ diffusion pathway from leaf surface to substomatal cavities. Since Xiong *et al*.^29^ suggested that the change of SS would affect *g*_sc_ in leaf anatomy, the enlargement of SS in this study would largely promote the increase of *g*_sc_ in both species.

In summary, our data showed a different effect of soil nitrogen addition levels on leaf anatomical characteristics in this study, which reached the maximum under MN treatment and decreased under the addition of HN, indicating a decreased positive effect from high soil nitrogen addition on plant metabolism. In addition, soil nitrogen addition could decrease the distance between the intercellular space and catalytic site of Rubisco (D_I-R_) and markedly could increase chloroplast size, finally facilitating CO_2_ diffusion in the liquid phase of mesophyll cells^6,68^. This phenomenon would be another important aspect to explain the effect of leaf anatomy on *g*_m_ during soil nitrogen addition.

## Conclusion

Soil nitrogen additions could enlarge CO_2_ diffusion conductance (both *g*_m_ and *g*_sc_) markedly in Manchurian ash and Mongolian oak in Changbai Mountains, but its promotion was dependent on the addition levels. Moderate soil nitrogen additions (≤46 kg N ha^−1^ a^−1^) increased *g*_m_ and *g*_sc,_ and they reached their maximum under the addition of 46 kg N ha^−1^ a^−1^, but this positive effect was weakened with the addition of high level of 69 kg N ha^−1^ a^−1^. The effects of soil nitrogen addition on *g*_m_ and *g*_sc_ mostly resulted from improvements in physiological traits, such as leaf N content and the activities of AQP and CA, and adjustments in anatomical characteristics, including *T*_leaf_, *T*_mes_, *S*_mes_ and stomatal opening status.

## Material and methods

### Plant material and experimental design

Five-year-old potted saplings of similar size in Manchurian ash and Mongolian oak were selected as the materials at the National Research Station of Changbai Mountain Forest Ecosystems of the Chinese Academy of Sciences located in Jilin province of northeast China (128°06′E, 42°24′N), and they were transplanted into individual pots in 2015, filled with 27 l soils collected from a broad-leaved Korean pine forest with a mean annual nitrogen forest deposition of 23 kg N ha^−1^ a^−1^. The roots in the pots were blocked from the outside soil by the pedestals placed under the pots. The volume of the pots was 29.28 L with a height of 30.0 cm and a diameter of 34.3 cm.

Four nitrogen addition levels were used to simulate nitrogen deposition intensities with the addition of no nitrogen (CK), low nitrogen (LN, 23 kg N ha^−1^ a^−1^), medium nitrogen (MN, 46 kg N ha^−1^ a^−1^) and high nitrogen (HN, 69 kg N ha^−1^ a^−1^). Urea solutions with different concentrations of nitrogen were sprayed into the pots once every other month from May to October in 2017. Five replicates were designed for each treatment, and all potted saplings were thoroughly watered daily to avoid water deficit. All measurements were carried out during summer growth season (July and August) to explore the changes in effects of soil nitrogen addition on *g*_m_ and *g*_sc_.

### Simultaneous gas exchange and chlorophyll fluorescence measurements

We simultaneously measured gas exchange and chlorophyll fluorescence on newly and fully expanded, sun-exposed leaves using an open-flow gas exchange system (Li-6400XT; Li-Cor Inc., Lincoln, NE, USA) equipped with an integrated fluorescence leaf chamber (Li-6400-40; Li-Cor). Leaves were fully light adopted under a saturated photosynthetic active photon flux density (PPFD) of 1200 µmol m^−2^ s^−1^ provided by Li-6400 with a 10: 90 blue: red light for 30 minutes, while leaf temperature and relative humidity and CO_2_ concentration in the leaf chamber were maintained at 25 °C and 60 ± 5% and 400 µmol CO_2_ mol^−1^ with a CO_2_ mixture, respectively. In addition, the gas flow rate was controlled at 300 μmol s^−1^ to ensure the adequate gas exchange. After stabilization to a steady state, gas exchange parameters, steady-state fluorescence (*F*_*s*_) and maximum fluorescence (*F*_*m*_′) were recorded.

The actual photochemical efficiency of photosystem II (*Φ*_PS_ II) was calculated according to Genty *et al*.^35^:

1 $${{\Phi }}_{{\rm{PSII}}}=\frac{({F{\prime} }_{m}-{F}_{{\rm{s}}})}{F{\prime} m}$$

The photosynthesis electron transport rate (*J*_f_) was calculated as follows:

2 $${J}_{{\rm{f}}}={{\Phi }}_{{\rm{PSII}}}\cdot PPFD\cdot \alpha \beta $$

where *α* is the total leaf absorptance and *β* is the partitioning of absorbed quantum between PS II and PS I.

In this study, light response curves (*A*_n_-PPFD curve) for controlled and nitrogen-addition saplings were also measured under a low O_2_ concentration (<1%) condition to correct the *αβ*, as it was equal to the slope of the relationship between *Φ*_PS_ II and 4*Φ*_CO2_ (the quantum efficiency of CO_2_ fixation)^36^. Values of *αβ* for different nitrogen treatments were shown in Table S1.

The mesophyll conductance (*g*_m_) was calculated using the ‘variable *J* method’ described in Harley *et al*.^37^:

3 $${g}_{{\rm{m}}}=\frac{{A}_{{\rm{n}}}}{{C}_{{\rm{i}}}-\frac{{\Gamma }^{\ast }({J}_{{\rm{f}}}+8({A}_{{\rm{n}}}+{R}_{{\rm{d}}}))}{{J}_{{\rm{f}}}-4({A}_{{\rm{n}}}+{R}_{{\rm{d}}})}}$$

where *A*_n_ is the net photosynthetic rate, *C*_i_ is the intercellular CO_2_ concentration, and these values were directly obtained from gas exchange measurements; Г^*^ represents the CO_2_ compensation point in the absence of respiration and *R*_d_ represents the mitochondrial respiration in the light.

Г^*^ and *R*_d_ were measured using the Laisk method, namely, 5 initial slopes of *A*_n_-*C*_i_ curves under low light and low CO_2_ concentrations were measured for Г^*^ and *R*_d_ estimations in this study^38,39^. In theory, three CO_2_ response curves obtained by varying CO_2_ concentrations from 150 to 40 µmol CO_2_ mol^−1^ under three PPFDs (150, 100 and 50 µmol m^−2^ s^−1^) would intersect with each other at a point, and the intersection point at *x*-axis and *y*-axis were considered to Г^*^ and *R*_d_, respectively. But in practice, these three linear regressions of the intersected *A*_n_-*C*_i_ curves formed a triangle range rather than a single point^40^ (Fig. S1), and the Г^*^ and *R*_d_ were calculated as the barycenter of the triangle formed by the intersection of the three lines at *x*-axis and *y*-axis, respectively, according to our previous published method^40^. The values of Г^*^ and *R*_d_ during soil nitrogen additions were shown in Table S2.

Stomatal conductance to CO_2_ (*g*_sc_, mol CO_2_ m^−2^ s^−1^) was calculated from the ratio of stomatal conductance to water (*g*_sw_, mol CO_2_ m^−2^ s^−1^) to 1.6 (i.e., *g*_sc_ = *g*_sw_/1.6), as *g*_sw_ was 1.6 times larger than *g*_sc_^41^.

### Measurement of leaf anatomical characteristics

After gas exchange measurements, we cut fifteen small leaf samples (4.0 mm × 1.5 mm) from five replicated leaves per treatment and fixed them in FAA (alcohol: formaldehyde: glacial acetic acid = 90: 5: 5) to measure leaf anatomical characteristics, mainly including leaf thickness (*T*_leaf_, μm), mesophyll and stomatal anatomical characteristics, such as the thickness of mesophyll cells between the two epidermal layers (*T*_mes_, μm) and the surface area of mesophyll cells exposed to intercellular space per unit leaf area (*S*_mes_, μm^2^ μm^−2^) calculated using formula (4), stomatal pore length (PL, μm) and width (PW, μm) at the centre of the stoma, which were detailed described in Zhu *et al*.^30^. Besides, we counted the number of stomata per unit leaf area to calculate the stomatal density (*D*_s_, N cm^−2^).

4 $${S}_{{\rm{mes}}}=\frac{{L}_{{\rm{mes}}}}{W}\cdot F$$

where the length of mesophyll cells exposes to intercellular space (*L*_mes_, μm) and the cross-sectional width (*W*, μm) are measured with Image J software. The curvature correction factor (*F*) was measured using the method described in the study of Evans *et al*.^42^ and Thain (1983)^43^, which was shown in Table S3.

We also calculated the gas- and liquid-phase CO_2_ diffusion conductance in mesophyll cells (i.e., *g*_ias_ and *g*_liq_) with leaf anatomical characteristics according to formulas (5) and (6) to assess their relationships with *g*_m_.

5 $${{\rm{g}}}_{{\rm{ias}}}=\frac{{D}_{{\rm{a}}}{f}_{{\rm{ias}}}}{\Delta {L}_{{\rm{ias}}}\varsigma }$$

6 $$\frac{1}{{{\rm{g}}}_{{\rm{liq}}}}=\left(\frac{1}{{{\rm{g}}}_{{\rm{cw}}}}+\frac{1}{{{\rm{g}}}_{{\rm{pl}}}}+\frac{1}{{{\rm{g}}}_{{\rm{ct}}}}+\frac{1}{{{\rm{g}}}_{{\rm{en}}}}+\frac{1}{{{\rm{g}}}_{{\rm{st}}}}\right)\frac{{A}_{{\rm{T}}}}{{A}_{{\rm{mes}}}}$$

where *D*_a_ (m^2^ s^−1^) is the diffusion coefficient for CO_2_ in the gas phase, Δ*L*_ias_ (μm) is taken as half *T*_mes_^26,44^, ϛ is the diffusion path tortuosity (m m^−1^) and *f*_ias_ (%) is the fraction of mesophyll volume occupied by the intercellular airspace. *g*_cw_, *g*_pl_, *g*_ct_, *g*_en_ and *g*_st_ are the partial conductance for the cell wall, plasmalemma, cytosol, chloroplast envelope and chloroplast stroma (m s^−1^), respectively. We used an estimation of 0.0035 m s^−1^ for the *g*_pl_ and *g*_en_ according to the previous studies^42,45^, and estimated *g*_cw_, *g*_ct_ and *g*_st_ using the formula of ${g}_{{\rm{i}}}=\frac{{r}_{{\rm{f}}}{D}_{{\rm{w}}}p}{\Delta {L}_{{\rm{i}}}}$ in the studies of Tomás *et al*.^26^ and Niinemets and Reichstein (2003)^44^, where *g*_i_ (m s^−1^) is either *g*_cw_, *g*_ct_ or *g*_st_, *r*_f_ is the dimensionless coefficient, *D*_w_ (m^2^ s^−1^) is the aqueous-phase volatile diffusion coefficient for CO_2_ (1.79 × 10^−9^ m^2^ s^−1^ at 25 °C), Δ*L*_i_ (m) is the diffusion path length, and *p* (m^3^ m^−3^) is the effective porosity. According to the previous studies of Tomás *et al*.^26^ and Niinemets and Reichstein (2003)^44^, *r*_f_ was valued as 1 for cell wall, and an estimate of *r*_f_ of 0.294 for *g*_ct_ and *g*_st_ in this study, *p* was taken as 1 for *g*_ct_ and *g*_st_, and 0.3 for cell walls, Δ*L*_i_ was valued as 5.0 × 10^−7^ (for cell wall, Δ*L*_cw_), 9.7 × 10^−8^ (for cytosol, Δ*L*_ct_) and 1.65 × 10^−6^ (for chloroplast stroma, Δ*L*_st_). *A*_mes_ (μm^2^) and *A*_T_ (μm^2^) are mesophyll surface area and total leaf surface area corrected for the actual area available for CO_2_ diffusions, respectively, calculated from the light microscope.

### Measurement of leaf N content

Leaves were picked and over-dried at 75 °C for 24 h to constant weight during gas exchange measuring periods and then ground using a mixer oscillating mill homogenizer (MM400, Retsch, Germany). Approximately 5.0 mg leaf samples were taken to measure leaf nitrogen content per area using a C N element analyser (Elementar vario MACRO, Element, Germany).

### Measurements of leaf aquaporin and carbonic anhydrase activities

For analysing the physiological mechanism of *g*_m_ and *g*_sc_ responses to soil nitrogen additions, we sampled fifteen fresh leaves per treatment to measure the activities of aquaporin (AQP) and carbonic anhydrase (CA) using enzyme-linked immunosorbent assay (ELISA)^46,47^. Solid-phase antibody was made using purified plant AQP (or CA) antibody. Then, combined with antibody labelled with horseradish peroxidase (HRP), AQP (or CA) was added to microtiter plate wells to become an antibody-antigen-enzyme-antibody complex. This complex became blue with 3,3′,5,5′-tetramethyl benzidine (TMB) substrate solution after complete washing. The optical density (OD) values were measured spectrophotometrically at a wavelength of 450 nm to compare with the standard curves to determine the activity of AQP (or CA) in the samples.

### Statistical analysis

SPSS 17.0 (SPSS Inc., Chicago, IL, USA) was used for one-way statistical analysis of normality and homogeneity of variance between nitrogen addition treatments (one-way ANOVA). Furthermore, regression analysis between *g*_m_ and leaf N content was also performed. Mean values were compared using the least significant difference (LSD) multiple comparison test at the 0.05 and 0.01 probability levels (*P* < 0.05 and *P* < 0.01) with Tukey′s honest significant difference (HSD) test.

## Supplementary information

Supplementary information.

## Data Availability

All data analysed during this study are included in this published article and its Supplementary Information files.
