# Supplementary material for: Effects of nitrogen additions on mesophyll and stomatal conductance in Manchurian ash and Mongolian oak
Source: Sci Rep. 2020 Jun 22;10:10038. doi: 10.1038/s41598-020-66886-x (PMC7308411; doi:10.1038/s41598-020-66886-x)
Supplement: Supplementary file 1 — Supplementary information. [file 41598_2020_66886_MOESM1_ESM.docx]

Supplementary Information for

**Effects of nitrogen additions on mesophyll and stomatal conductance in Manchurian ash and Mongolian oak**

Kai Zhu^1, 2^, Anzhi Wang^1^, Jiabing Wu^1^, Fenghui Yuan^1*^, Dexin Guan^1*^, Changjie Jin^1^, Yushu Zhang^3^, Chunjuan Gong^1, 2^

1 Key Laboratory of Forest Ecology and Management, Institute of Applied Ecology, Chinese Academy of Sciences, Shenyang 110016, China

2 University of Chinese Academy of Sciences, Beijing 100049, China

3 The Institute of Atmospheric Environment, China Meteorological Administration, Shenyang 110016, China

*Corresponding author:

Fenghui Yuan, E-mail: fhyuan@iae.ac.cn, Tel: 86-24-83970336;

Dexin Guan, E-mail: dxguan@iae.ac.cn, Tel: 86-24-88087869

Permanent address:

72 Wenhua Road Shenhe District, Institute of Applied Ecology, Shenyang, China

This file includes:

Three Tables. S1 to S3 and one Figure. S1.

**Table S1.** Values of *αβ* for different nitrogen treatments in both species. All data were means ± SE (n = 5). Different lowercase letters (a, b, c) indicated significant differences between nitrogen addition treatments at *P* < 0.05. CK, the control; LN, low nitrogen addition; MN, medium nitrogen addition; HN, high nitrogen addition.

| Treatments | Manchurian ash | Mongolian oak |
| --- | --- | --- |
| CK | 0.39±0.01^d^ | 0.37±0.02^d^ |
| LN | 0.42±0.01^abc^ | 0.38±0.03^cd^ |
| MN | 0.40±0.01^bcd^ | 0.41±0.01^abc^ |
| HN | 0.40±0.01^cd^ | 0.39±0.01^bcd^ |

**Table S2.** Values of CO_2_ compensation point in the absence of respiration (*Г*^*^, μmol mol^-1^) and dark respiration (*R*_d_, μmol m^-2^ s^-1^) for different nitrogen addition treatments in both species. All data were means ± SE (n = 5). Different lowercase letters (a, b, c) indicated significant differences between nitrogen addition treatments at *P* < 0.05. CK, the control; LN, low nitrogen addition; MN, medium nitrogen addition; HN, high nitrogen addition.

|  | Manchurian ash | | Mongolian oak | |
| --- | --- | --- | --- | --- |
|  | *Γ**  (μmol mol^-1^) | *R*_d_  (μmol m^-2^ s^-1^) | *Γ**  (μmol mol^-1^) | *R*_d_  (μmol m^-2^ s^-1^) |
| CK | 47.1 ± 5.6^a^ | 1.00 ± 0.05^ab^ | 50.1 ± 3.6^b^ | 1.00 ± 0.02^c^ |
| LN | 42.5 ± 3.1^a^ | 1.05 ± 0.06^a^ | 40.7 ± 2.8^c^ | 1.36 ± 0.06^a^ |
| MN | 47.0 ± 3.8^a^ | 0.94 ± 0.04^b^ | 58.4 ± 3.5^a^ | 0.92 ± 0.04^c^ |
| HN | 46.6 ± 2.8^a^ | 0.98 ± 0.06^ab^ | 49.6 ± 1.9^b^ | 1.14 ± 0.05^b^ |

**Table S3.** The *F* values for different nitrogen treatments in both species. CK, the control; LN, low nitrogen addition; MN, medium nitrogen addition; HN, high nitrogen addition.

| Treatments | Manchurian ash | Mongolian oak |
| --- | --- | --- |
| CK | 1.34 | 1.35 |
| LN | 1.36 | 1.35 |
| MN | 1.31 | 1.33 |
| HN | 1.37 | 1.35 |








**Fig. S1.** Linear regressions of the net CO_2_ assimilation rate (*A*_n_) *vs* the intercellular CO_2_ concentrations (*C*_i_) at three PPFD values of 50 (black squares), 100 (black circles) and 150 (black triangles) μmol m^-2^ s^-1^ for controlled (CK), low (LN), medium (MN) and high nitrogen addition (HN) leaves in Manchurian ash (Ⅰ) and Mongolian oak (Ⅱ). The CO_2_ compensation point in the absence of respiration (*Г*^*^) and mitochondrial respiration in the light (*R*_d_) were calculated as the barycenter of the triangle formed by the intersection of the three lines at *x*-axis and *y*-axis, respectively. Data were presented as means ± SE (n = 5).
